# Sodium Levels and Outcomes in Patients With Metastatic Renal Cell Carcinoma Receiving Nivolumab

**DOI:** 10.1001/jamanetworkopen.2023.45185

**Published:** 2023-11-27

**Authors:** Martina Catalano, Sara Elena Rebuzzi, Marco Maruzzo, Ugo De Giorgi, Sebastiano Buti, Luca Galli, Giuseppe Fornarini, Paolo Andrea Zucali, Giuseppe Procopio, Silvia Chiellino, Michele Milella, Fabio Catalano, Stefania Pipitone, Riccardo Ricotta, Mariella Sorarù, Veronica Mollica, Marianna Tudini, Lucia Fratino, Veronica Prati, Orazio Caffo, Francesco Atzori, Franco Morelli, Giuseppe Prati, Franco Nolè, Francesca Vignani, Alessia Cavo, Marilena Di Napoli, Andrea Malgeri, Emanuele Naglieri, Alessio Signori, Giuseppe Luigi Banna, Pasquale Rescigno, Lorenzo Antonuzzo, Giandomenico Roviello

**Affiliations:** 1 Department of Health Sciences, Section of Clinical Pharmacology and Oncology, University of Firenze, Firenze, Italy; 2 Medical Oncology Unit, Ospedale San Paolo, Savona, Italy; 3 Department of Internal Medicine and Medical Specialties, University of Genoa, Genoa, Italy; 4 Oncology 1 Unit, Department of Oncology, Istituto Oncologico Veneto IOV–IRCCS, Padova, Italy; 5 Department of Medical Oncology, IRCCS Istituto Romagnolo per lo Studio dei Tumori Dino Amadori, Meldola, Italy; 6 Medical Oncology Unit, University Hospital of Parma, Parma, Italy; 7 Department of Medicine and Surgery, University of Parma, Parma, Italy; 8 Medical Oncology Unit 2, Azienda Ospedaliero-Universitaria Pisana, Pisa, Italy; 9 Medical Oncology Unit 1, IRCCS Ospedale Policlinico San Martino of Genova, Genova, Italy; 10 Department of Biomedical Sciences, Humanitas University, Pieve Emanuele, Milano, Italy; 11 Department of Oncology, IRCCS Humanitas Research Hospital, Rozzano, Milan, Italy; 12 SS Oncologia Medica Genitourinaria, Fondazione IRCCS Istituto Nazionale dei Tumori, Milano, Italy; 13 Medical Oncology Unit, IRCCS Policlinico San Matteo, Pavia, Italy; 14 Section of Innovation Biomedicine–Oncology Area, Department of Engineering for Innovation Medicine, University of Verona and Verona University and Hospital Trust, Verona, Italy; 15 Medical Oncology Unit, Department of Oncology and Hematology, University Hospital of Modena, Modena, Italy; 16 Oncology Unit, IRCCS MultiMedica, Sesto san Giovanni, Milano, Italy; 17 U.O. Oncologia, Ospedale di Camposampiero, Camposampiero, Italy; 18 Medical Oncology, IRCCS Azienda Ospedaliero-Universitaria di Bologna, Bologna, Italy; 19 Medical Oncology, St Salvatore Hospital, L’Aquila, Italy; 20 Department of Medical Oncology, Centro di Riferimento Oncologico di Aviano CRO-IRCCS, Aviano, Italy; 21 Medical Oncology Unit, ASL CN 2, Alba-Bra, Italy; 22 Department of Medical Oncology, Santa Chiara Hospital, Trento, Italy; 23 Medical Oncology Department, University Hospital, University of Cagliari, Cagliari, Italy; 24 Medical Oncology Department, Casa Sollievo Della Sofferenza Hospital, IRCCS, San Giovanni Rotondo, Italy; 25 Department of Oncology and Advanced Technologies AUSL–IRCCS Reggio Emilia, Reggio Emilia, Italy; 26 Medical Oncology Division of Urogenital & Head & Neck Tumors, IEO, European Institute of Oncology IRCCS, Milano, Italy; 27 Division of Medical Oncology, Ordine Mauriziano Hospital, Torino, Italy; 28 Oncology Unit, Villa Scassi Hospital, Genova, Italy; 29 Department of Urology and Gynecology, Istituto Nazionale Tumori IRCCS Fondazione G. Pascale, Naples, Italy; 30 Department of Medical Oncology, Fondazione Policlinico Campus Bio-Medico, Roma, Italy; 31 Division of Medical Oncology, IRCCS Istituto Tumori Giovanni Paolo II, Bari, Italy; 32 Department of Health Sciences, Section of Biostatistics, University of Genova, Genova, Italy; 33 Portsmouth Hospitals University NHS Trust, Portsmouth, United Kingdom; 34 Faculty of Science and Health, School of Pharmacy and Biomedical Sciences, University of Portsmouth, Portsmouth, United Kingdom; 35 Candiolo Cancer Institute, FPO-IRCCS, Candiolo, Turin, Italy; 36 Medical Oncology, Department of Experimental and Clinical Medicine, University of Florence, Florence, Italy

## Abstract

**Importance:**

Low sodium levels have been associated with negative outcomes among patients with metastatic renal cell carcinoma (mRCC) receiving therapies other than immune checkpoint inhibitors (ICIs).

**Objective:**

To investigate the role of natremia in patients with mRCC receiving nivolumab as a second-line or subsequent therapy.

**Design, Setting, and Participants:**

In this retrospective cohort study, the clinical and biochemical data of patients with mRCC receiving nivolumab were collected from October 2015 to November 2019 as part of a multicenter Italian study. Data analysis was performed from February to March 2023.

**Exposure:**

Nivolumab was administered intravenously at a dose of 3 mg/kg every 2 weeks and, since May 2018, at a fixed dose of 240 mg every 2 weeks or 480 mg every 4 weeks. Patients were divided into 2 groups according to their median serum sodium value (<140 or ≥140 mEq/L).

**Main Outcomes and Measures:**

The primary outcomes were the associations of pre-ICI and post-ICI sodium levels with overall survival (OS), progression-free survival (PFS), objective response rate, and disease control rate (DCR). The Kaplan-Meier method was used to estimate PFS and OS, and differences between groups were compared using the log-rank test.

**Results:**

A total of 401 patients with mRCC receiving nivolumab as second-line therapy were evaluated, and 355 eligible patients (median [range] age, 76 [44-84] years; 258 male patients [72.7%]) were included in the final cohort. Among patients with pre-ICI sodium greater than or equal to 140 mEq/L compared with those with sodium less than 140 mEq/L, the median PFS was 9.3 months (95% CI, 6.5-11.5 months) vs 7.4 months (95% CI, 4.6-10.1 months; *P* = .90), and the median OS was 29.2 months (95% CI, 21.8-35.9 months) vs 20.0 months (95% CI, 14.1-26.8 months; *P* = .03). Patients with post-ICI sodium values greater than or equal to 140 mEq/L had longer PFS (11.1 months [95% CI, 8.5-1.5 months] vs 5.1 months [95% CI, 4.1-7.5 months]; *P* = .01) and OS (32.9 months [95% CI, 25.1-42.6 months] vs 17.1 months [95% CI, 12.6-24.5 months]; *P* = .006) compared with patients with sodium values less than 140 mEq/L. Patients with both pre-ICI and post-ICI sodium values greater than or equal to 140 mEq/L exhibited a significant improvement in clinical outcomes compared with those with a value less than 140 mEq/L (PFS, 11.5 months [95% CI, 8.8-16.4 months] vs 5.8 months [95% CI, 4.4-8.3 months]; *P* = .008); OS, 37.6 months [95% CI, 29.0-49.9 months] vs 19.4 months [95% CI, 14.1-24.5 months]; *P* = .01). Moreover, sodium levels greater than or equal to 140 mEq/L were associated with significantly better DCR than lower sodium levels.

**Conclusions and Relevance:**

In this retrospective cohort study of patients with mRCC receiving nivolumab, sodium values greater than or equal to 140 mEq/L, both before and/or after ICI, were associated with better OS and PFS, as well as a higher DCR, compared with levels less than 140 mEq/L. These findings suggest that sodium levels may be associated with survival outcomes in patients with mRCC and may have potential use as variables to consider in patients’ risk scores.

## Key Points

**Question:**

Are sodium levels associated with outcomes in patients with metastatic renal cell carcinoma (mRCC) treated with nivolumab monotherapy?

**Findings:**

In this cohort study of 355 patients with mRCC treated with nivolumab, lower sodium levels (<140 mEq/L) were associated with shorter overall survival and progression-free survival and lower disease control rate compared with sodium levels greater than or equal to 140 mEq/L.

**Meaning:**

These findings suggest that serum sodium level may be associated with survival outcomes in patients with mRCC receiving immunotherapy and has potential use as a variable to consider in patients’ risk scores.

## Teaser

This cohort study examines the association of serum sodium levels with clinical outcomes in patients with metastatic renal cell carcinoma (RCC) receiving nivolumab.

## Introduction

Renal cell carcinoma (RCC) comprises approximately 3% of all malignant tumors in adulthood, with approximately 430 000 new cases and 179 368 deaths worldwide in 2020.^1^ Over the past few decades, the treatment landscape for metastatic RCC (mRCC) has undergone major transformations, with the inclusion of immunotherapeutic agents and targeted receptor tyrosine kinase inhibitors (TKIs), resulting in a gradual improvement in outcomes.^2,3,4^ For patients with intermediate-risk and poor-risk disease, double immune checkpoint inhibitor (ICI) therapy with nivolumab plus ipilimumab serves as a viable first-line option.^5^ In specific cases, single-agent immunotherapy or TKI may be considered.^5^ Despite initial favorable response rates, acquired resistance is nearly universal.^6^ Identifying the most suitable sequence of postprogression treatments presents a clinical difficulty, because it greatly depends on such variables as prior treatment, the extent of disease, tumor characteristics, and the patient’s medical background and current health condition.^7^ The selection of the most suitable therapy for mRCC is primarily based on clinical features and biochemical examination. However, there is a pressing need to explore potential new prognostic markers.^8,9,10^

Previous studies^11,12,13^ have shown that serum sodium levels can serve as a prognostic marker for several diseases, including malignant tumors. Hyponatremia, defined as a serum sodium level below 135 mEq/L (to convert to millimoles per liter, multiply by 1), is an independent prognostic factor for various solid malignant tumors including RCC.^14,15,16,17^ It has been associated with a poorer outcomes and shorter cancer-specific survival in patients with mRCC treated with several types of drugs, including TKIs, mammalian target of rapamycin–targeted agents,^18^ interleukin-2, and interferon-α.^19^ However, to our knowledge, no associations with ICI in mRCC have been previously reported.

The exact mechanisms leading to hyponatremia in patients with RCC remain unclear. Although an ectopic and inappropriate production of antidiuretic hormone (ADH) is uncommon in RCC compared with other tumor types, it may partially explain the occurrence of hyponatremia, as well as a postnephrectomy renal dysfunction.^17,20^ In addition, hyponatremia can occur as a consequence of gastrointestinal, neurological, or endocrinological adverse events during ICI treatment.

Although serum sodium levels are routinely measured at baseline and during cancer treatment, the role of natremia in patients with mRCC receiving ICIs has not been thoroughly investigated. Therefore, in this study, we conducted a multicenter retrospective analysis to assess the sodium values on the response rate and survival outcomes in pretreated patients with mRCC receiving nivolumab as second-line or subsequent therapy.

## Methods

### Patients and Treatment

In this cohort study, we retrospectively analyzed the clinical data of all consecutive patients with mRCC who received nivolumab as second-line or subsequent therapy from October 2015 to November 2019 at several Italian oncology centers (subanalysis of the Meet-URO 15 study).^21^ The inclusion criterion for this subanalysis was the availability of serum sodium values at baseline (referred to as pre-ICI) and approximately 4 weeks after the first administration of ICI therapy (referred to as post-ICI). We recorded various demographic and clinical data for all patients, including histologic RCC type, Karnofsky–Performance Status (PS) score, risk group based on International Metastatic Renal Cell Carcinoma Database (IMDC) criteria, metastatic sites, first-line therapy used, and serum sodium values.

Nivolumab was initially administered intravenously at a dose of 3 mg/kg every 2 weeks and, since May 2018, at the fixed dose of 240 mg every 2 weeks, or 480 mg every 4 weeks, according to local clinical practice, until disease progression or unacceptable toxic levels. Ethical approval for this study was obtained from the Ethics Regional Ethical Committee of Liguria, and written informed consent was obtained from all participating patients. This report follows the Strengthening the Reporting of Observational Studies in Epidemiology (STROBE) reporting guidelines for cohort studies.

### Assessment

Serum sodium levels were assessed as a routine laboratory measurement at baseline, within 10 days of starting the treatment, and before each therapy cycle. Normal natremia was defined as a serum sodium level greater than or equal to 135 and less than or equal to 145 mEq/L according to the laboratory’s reference range. Response evaluation was performed every 3 months using spiral computed tomography and assessed according to the Response Evaluation Criteria in Solid Tumor version 1.1.^22^ The efficacy of the treatment was evaluated in terms of overall survival (OS) and progression-free survival (PFS). Adverse events occurring during nivolumab administration were monitored by the investigators and were reported. Immune-related adverse events were evaluated using the Common Terminology Criteria of Adverse Events version 5.0.^23^ Several variables, including age, sex, histologic profile, previous surgery, Karnofsky-PS score, IMDC score, the number of metastatic sites, and the levels of serum sodium at before and after the start of treatment, were assessed for their associations with outcomes.

### Outcome Variables

The objective of this study was to assess the association of sodium levels (pre-ICI and/or post-ICI) with the efficacy and survival outcomes of patients with mRCC who received nivolumab as second-line or subsequent therapy. To accomplish this, patients were divided into 2 groups according to their median serum sodium levels. The primary outcomes evaluated were PFS, defined as the time from initiation of treatment to disease progression or death, and OS, defined as the time elapsed between treatment initiation and death from any cause. The secondary end points included the disease control rate (DCR), which indicates the proportion of patients achieving complete response, partial response, or stable disease, as well as the objective response rate, representing the proportion of patients achieving complete response or partial response.

### Statistical Analysis

Data analysis was performed from February to March 2023. Descriptive statistics were used to analyze the demographic and tumor characteristics of the study population. Continuous variables were presented as medians with ranges indicating the minimum and maximum values, whereas categorical variables were expressed as numbers and percentages. The Kaplan-Meier method was used to estimate PFS and OS, and differences between groups were compared using the log-rank test. The Cox proportional hazard model was used to calculate the hazard ratios (HRs) and their corresponding 2-sided 95% CIs.

In the univariate analysis, potential factors associated with PFS and OS were assessed, and variables with *P* ≤ .05 were selected for inclusion in the multivariate analysis. The multivariate Cox regression model was adjusted for potential confounding factors, such as IMDC score, Karnofsky-PS score, previous nephrectomy, and pre-ICI and post-ICI serum sodium levels. To analyze secondary outcomes, the variables were dichotomized, and the Fisher exact test was used to analyze the association of the dichotomized serum sodium values with clinical and biochemical variables. The statistical analysis was performed using Stata statistical software version 9.1 (StataCorp). Statistical significance was set at 2-sided *P* < .05.

## Results

### Patients’ Characteristics

Of a total of 401 patients with mRCC treated with nivolumab as second or subsequent line of therapy, 355 were eligible and included in the study. The median (range) age of the patients was 76 (44-84) years. Among the included patients, 258 (72.7%) were male. Most patients (306 patients [86.7%]) had RCC with a clear cell histologic profile, and 279 (78.6%) were classified as having intermediate-poor risk according to the IMDC criteria. Almost all patients (308 patients [87.0%]) had a Karnofsky-PS score of 80% or higher. Visceral metastases were detected in 326 patients (91.8%), bone metastases were detected in 120 patients (33.8%), and lymph node metastases were observed in 202 patients (56.9%). The first-line treatments received by the patients included sunitinib (220 patients [62.0%]), pazopanib (127 patients [36.6%]), or other treatment options (8 patients [2.5%]). Nivolumab was administered to 245 patients (69.0%) as a second-line treatment, to 77 patients (21.7%) as a third-line treatment, and to 33 patients (9.2%) as a fourth-line or later treatment. Nephrectomy had been previously performed for 313 patients (88.2%) (Table 1). The sodium levels ranged from 129 and 149 mEq/L, with a median value of 140 mEq/L.

**Table 1.** Patients’ Baseline Characteristics^a^

Characteristic	Patients, No. (%) (N = 355)
Age, median (range), y	76 (44-84)
Sex	
Male	258 (72.7)
Female	97 (27.3)
Clear-cell renal cell carcinoma histologic profile	306 (86.7)
Previous nephrectomy	313 (88.2)
Karnofsky–Performance Status score ≥80%	308 (87.0)
International Metastatic Renal Cell Carcinoma Database score of intermediate-poor	279 (78.6)
Sites of metastases	
Lymph nodes	202 (56.9)
Visceral	326 (91.8)
Bone	120 (33.8)
First-line therapy	
Sunitinib	220 (62.0)
Pazopanib	127 (35.6)
Other	8 (2.5)
Nivolumab line	
Second line	245 (69.0)
Third line	77 (21.7)
Fourth line or later	33 (9.2)
Sodium level before immune checkpoint inhibitor therapy, median (range), mEq/L	140 (129-149)

SI conversion factor: To convert sodium to millimoles per liter, multiply by 1.

^a^ Evaluation was performed approximately 30 days after the start of immune checkpoint inhibitor therapy.

eTables 1, 2, and 3 in Supplement 1 present the baseline characteristics of patients according to their median sodium level (<140 or ≥140 mEq/L) at pre-ICI and post-ICI evaluations. In the pretreatment evaluation, 194 patients (54.6%) had a natremia level greater than or equal to 140 mEq/L, whereas 161 patients (45.4%) had a level less than 140 mEq/L; 26 patients had pretreatment sodium levels below the lower limit of the laboratory range (≤135 mEq/L). There were no statistically significant differences observed in demographic and clinical features between patients with serum sodium less than 140 mEq/L and those with sodium greater than or equal to 140 mEq/L in the pre-ICI evaluation.

At the post-ICI evaluation, 172 patients (48.4%) had a serum sodium level greater than or equal to 140 mEq/L, whereas 183 patients (51.6%) had a level less than 140 mEq/L. Like the pre-ICI evaluation, no other statistically significant differences were recorded. Patients with serum sodium greater than or equal to 140 mEq/L at both pre-ICI and post-ICI assessment did not differ from patients with at least 1 natremia value less than 140 mEq/L in the pre-ICI or post-ICI evaluation.

### Efficacy and Response Outcomes

**Figure 1.**
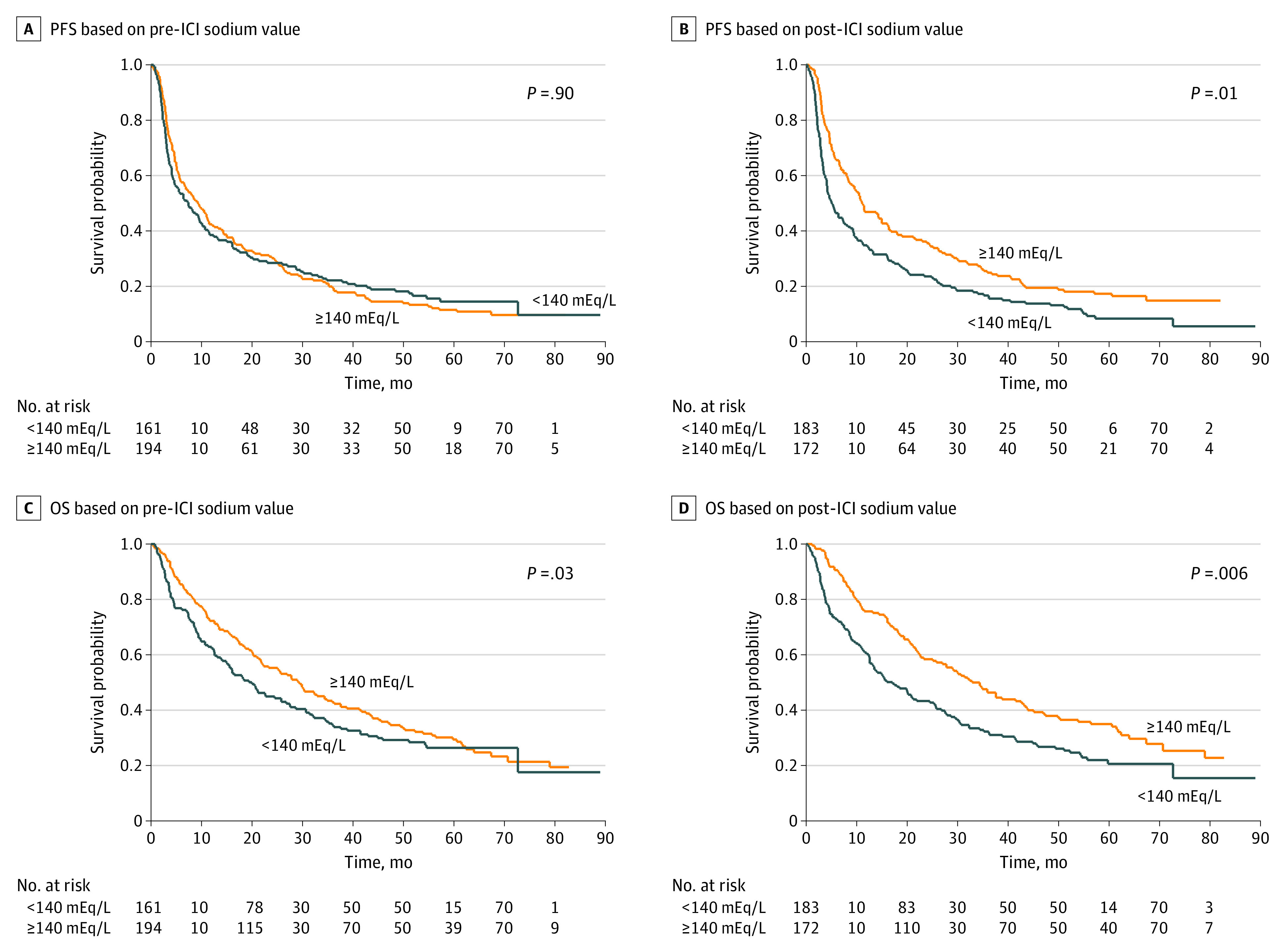
Kaplan-Meier Survival Estimate According to Serum Sodium Values Graphs show progression-free survival (PFS) by pre–immune checkpoint inhibitor (ICI) sodium values (A), PFS by post-ICI sodium values (B), overall survival (OS) by pre-ICI sodium values (C), and OS by post-ICI sodium values (D). To convert sodium to millimoles per liter, multiply by 1.

**Figure 2.**
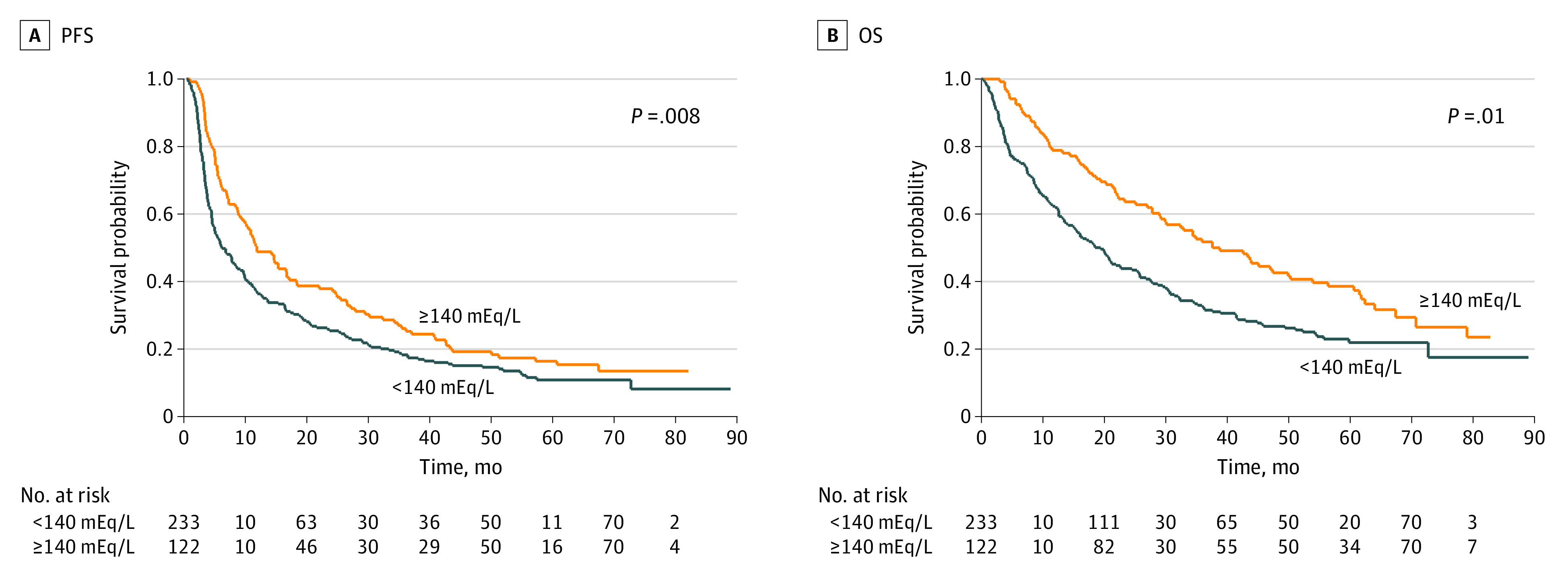
Kaplan-Meier Survival Estimate According to Serum Sodium Values Both Before and After Immune Checkpoint Inhibitor Therapy Graphs show progression-free survival (PFS) (A) and overall survival (OS) (B). To convert sodium to millimoles per liter, multiply by 1.

The analysis of efficacy outcomes and the best response was conducted according to median serum sodium levels (<140 or ≥140 mEq/L) and the assessment time (pre-ICI and post-ICI). At the time of data cutoff (May 2023), with a median (range) follow-up of 22.1 (1.0-89.0) months, 306 patients had experienced disease progression, and 253 had died. The median OS was significantly longer among patients with high (≥140 mEq/L) pretreatment sodium levels compared with patients with low (<140 mEq/L) sodium levels (29.2 [95% CI, 21.8-35.9 months] vs 20.0 months [95% CI, 14.1-26.8 months]; *P* = .03). There were no significant differences observed in PFS (9.3 months [95% CI, 6.5-11.5 months] vs 7.4 months [95% CI, 4.6-10.1 months]; *P* = .90). At the post-ICI evaluation, patients with serum sodium levels greater than or equal to 140 mEq/L had longer median PFS (11.1 months [95% CI, 8.5-1.5 months] vs 5.1 months [95% CI, 4.1-7.5 months]; *P* = .01) and OS (32.9 months [95% CI, 25.1-42.6 months] vs 17.1 months [95% CI, 12.6-24.5 months]; *P* = .006) compared with patients with levels less than 140 mEq/L (Figure 1). The same trend was observed in patients with natremia greater than or equal to 140 mEq/L at both pre-ICI and post-ICI evaluation compared with patients with at least 1 natremia level less than 140 mEq/L (PFS, 11.5 months [95% CI, 8.8-16.4 months] vs 5.8 months [95% CI, 4.4-8.3 months]; *P* = .008; OS, 37.6 months [95% CI, 29.0-49.9 months] vs 19.4 months [95% CI, 14.1-24.5 months]; *P* = .01) (Figure 2 and eTable 4 in Supplement 1).

No differences were observed in the objective response rate between patients with serum sodium levels above or below 140 mEq/L at pre-ICI, post-ICI, and at both pre-and post-ICI evaluation. However, patients with serum sodium levels greater than or equal to 140 mEq/L at the post-ICI evaluation and at both pre-ICI and post-ICI evaluation had a better DCR compared with patients with lower sodium levels (eTable 4 in Supplement 1).

In the univariate survival analysis, the following factors were found to be associated with PFS: previous nephrectomy (HR, 0.56; 95% CI, 0.40-0.78; *P* = .005), Karnofsky-PS score greater than or equal to 80% (HR, 0.39; 95% CI, 1.28-0.54; *P* = .008), IMDC intermediate-poor risk score (HR, 1.71; 95% CI, 1.29-2.28; *P* = .005), bone metastasis (HR, 1.49; 95% CI, 1.18-1.88; *P* = .007), post-ICI serum sodium greater than or equal to 140 mEq/L (HR, 0.67; 95% CI, 0.54-0.84; *P* = .003), and serum sodium greater than or equal to 140 mEq/L at both pre-ICI and post-ICI evaluation (HR, 0.72; 95% CI, 0.57-0.92; *P* = .009). The following factors were significantly associated with OS: previous surgery (HR, 0.43; 95% CI, 0.30-0.61; *P* = .004), Karnofsky-PS score greater than or equal to 80% (HR, 0.28; 95% CI, 0.20-0.39; *P* < .001), IMDC intermediate-poor risk score (HR, 2.44; 95% CI, 1.72-3.46; *P* < .001), bone metastasis (HR, 1.62; 95% CI, 1.26-2.09; *P* = .003), post-ICI serum sodium greater than or equal to 140 mEq/L (HR, 0.66; 95% CI, 0.51-0.84; *P* = .005), pre-ICI serum sodium greater than or equal to 140 mEq/L (HR, 0.72; 95% CI, 0.54-0.90; *P* = .04), and serum sodium greater than or equal to 140 mEq/L at both pre-ICI and post-ICI evaluation (HR, 0.62; 95% CI, 0.48-0.82; *P* = .006).

In the multivariate analysis, all these factors maintained a statistically significant association with both PFS and OS, except for previous nephrectomy, which did not show a significant association with PFS. Refer to Tables 2 and 3 for the detailed results of the univariate and multivariate analyses.

**Table 2.** Univariate Analysis for Progression-Free Survival and Overall Survival

Variable	HR (95% CI)	*P* value
Progression-free survival		
Age >75 y	0.92 (0.68-1.24)	.91
Male sex	0.99 (0.77-1.27)	.90
Histologic profile, clear cell renal cell carcinoma	0.91 (0.66-1.26)	.61
Previous nephrectomy	0.56 (0.40-0.78)	.005
Karnofsky–Performance Status score ≥80%	0.39 (0.28-0.54)	<.001
International Metastatic Renal Cell Carcinoma Database score, intermediate-poor	1.71 (1.29-2.28)	<.001
Lymph node metastases	0.99 (0.79-1.25)	.90
Visceral metastases	0.95 (0.63-1.43)	.82
Bone metastases	1.49 (1.18-1.88)	.007
First-line therapy, sunitinib vs pazopanib	1.13 (0.89-1.43)	.31
Nivolumab line, second vs third or later	0.95 (0.75-1.21)	.72
Pre-ICI sodium ≥140 mEq/L	0.98 (0.79-1.23)	.90
Post-ICI sodium ≥140 mEq/L	0.67 (0.54-0.84)	.003
Pre-ICI and post-ICI sodium ≥140 mEq/L	0.72 (0.57-0.92)	.009
Overall survival		
Age >75 y	0.93 (0.67-1.30)	.70
Male sex	0.93 (0.71-1.24)	.61
Histologic profile, clear-cell renal cell carcinoma	1.01 (0.71-1.46)	.90
Previous nephrectomy	0.43 (0.30-0.61)	.004
Karnofsky–Performance Status score ≥80%	0.28 (0.20-0.39)	<.001
International Metastatic Renal Cell Carcinoma Database score, intermediate-poor	2.44 (1.72-3.46)	<.001
Lymph node metastases	1.09 (0.84-1.39)	.50
Visceral metastases	0.87 (0.56-1.37)	.61
Bone metastases	1.62 (1.26-2.09)	.003
First-line therapy, sunitinib vs pazopanib	0.94 (0.73-1.23)	.71
Nivolumab line, second vs third or later	0.91 (0.70-1.18)	.50
Pre-ICI sodium ≥140 mEq/L	0.72 (0.54-0.90)	.04
Post-ICI sodium ≥140 mEq/L	0.66 (0.51-0.84)	.005
Pre-ICI and post-ICI sodium ≥140 mEq/L	0.62 (0.48-0.82)	.006

Abbreviations: HR, hazard ratio; ICI, immune checkpoint inhibitor.

SI conversion factor: To convert sodium to millimoles per liter, multiply by 1.

**Table 3.** Multivariate Analysis for Progression-Free Survival and Overall Survival

Variable	HR (95% CI)	*P* value
Progression-free survival		
Previous nephrectomy	0.87 (0.55-1.21)	.15
Karnofsky–Performance Status score ≥80%	0.45 (0.31-0.78)	<.001
International Metastatic Renal Cell Carcinoma Database score of intermediate-poor	1.75 (1.22-2.25)	<.001
Bone metastases	1.20 (1.10-1.62)	.03
Overall survival		
Previous nephrectomy	0.68 (0.45-0.81)	<.001
Karnofsky–Performance Status score ≥80%	0.50 (0.35-0.72)	<.001
International Metastatic Renal Cell Carcinoma Database score of intermediate-poor	2.01 (1.69-3.23)	<.001
Bone metastases	1.28 (1.05-1.75)	.03
Pre–immune checkpoint inhibitor therapy sodium ≥140 mEq/L	0.78 (0.60-0.89)	.04

Abbreviation: HR, hazard ratio.

SI conversion factor: To convert sodium to millimoles per liter, multiply by 1.

## Discussion

The treatment landscape for metastatic RCC has evolved rapidly, particularly with the introduction of frontline immunotherapy, leading to improved patient outcomes.^24^ However, there is limited knowledge about selecting optimal therapies for patients who develop resistance in the second or subsequent lines of treatment after progression. Since 2015, nivolumab monotherapy has become the standard of care for patients whose disease progressed while they were receiving antivascular endothelial growth factor receptor treatment, according to the CheckMate 025 trial results.^25^ Nivolumab demonstrated superiority over everolimus in terms of overall response rate, 5-year PFS, OS, and quality of life.^25^ Currently, it remains a therapeutic option for selected patients receiving TKI monotherapy as a first-line treatment in favorable-risk patients. Recently, the Meet-URO 15 study^21^ investigated the prognostic role of clinical factors and inflammatory indices in pretreated patients with mRCC receiving second-line or subsequent nivolumab, providing a tool (Meet-URO score) that has higher accuracy than the IMDC alone and is easily applicable in clinical practice.

To our knowledge, this cohort study is the first to evaluate the association of sodium levels with outcomes in patients with mRCC receiving the ICI nivolumab as second-line or subsequent therapy. Our findings revealed that a pre-ICI sodium level greater than or equal to 140 mEq/L was associated with a significant improvement in OS. Furthermore, patients with sodium levels greater than or equal to 140 mEq/L after starting treatment showed longer PFS and OS. In addition, patients with sodium levels greater than or equal to 140 mEq/L at both pre-ICI and post-ICI evaluation had longer PFS and OS compared with those with at least a sodium level less than 140 mEq/L. Notably, patients with sodium levels greater than or equal to 140 mEq/L at the post-ICI evaluation and both pre-ICI and post-ICI evaluation demonstrated a better DCR. These results are consistent with those of our recent study,^26^ which showed that lower, but in range (≥135 and <140 mEq/L), sodium levels were associated with worse PFS and OS in patients with mRCC receiving TKIs as first-line therapy.

Serum sodium levels are frequently measured in clinical practice, but their prognostic value in mRCC is not clearly defined. Previous evidence has indicated an association of hyponatremia with poor outcomes in various cancers including RCC.^14,15,16,17^ Hyponatremia has been shown to be associated with negative outcomes both in patients with localized RCC and in patients with mRCC receiving several types of drugs, including low-dose interleukin-2, interferon-α, mammalian target of rapamycin inhibitors, or TKIs.^18,19^ However, there is a lack of data regarding the prognostic role of hyponatremia in patients with RCC receiving ICIs.

The causes of hyponatremia in patients with cancer can vary, including the syndrome of inappropriate ADH release, disturbances in the renin-angiotensin-aldosterone axis, poor adrenal gland function, and mild renal impairment due to nephrectomy.^27,28,29^ As previously reported,^30^ hyponatremia is a common electrolyte disorder after major urologic operations, including partial or radical nephrectomy, especially in patients with high-risk perioperative characteristics. In patients receiving nivolumab, hyponatremia can be attributed to hypovolemia, syndrome of inappropriate ADH secretion, or endocrinopathies related to ICIs, such as hypophysitis, primary adrenal insufficiency, and hypothyroidism.^31,32^ Hyponatremia, regardless of its causes, should be recognized as an important warning sign of poor outcomes in patients with cancer. Furthermore, serum electrolyte levels, such as sodium, can indicate changes in the patients’ nutritional status, which could affect outcomes. Recent evidence suggests that a lower albumin level (a recognized marker of nutritional status), when combined with lymphocyte count in a Prognostic Nutritional Index, serves as a negative prognostic factor for patients with RCC, because it is associated with tumor progression and reduced survival.^33^

Our study provided evidence of an association of higher sodium levels with a better response to ICI. Similarly, previous studies^18^ reported a higher likelihood of primary resistance to targeted therapy or lower tolerance in patients with baseline hyponatremia. The role of serum sodium in the survival and treatment response of patients with mRCC needs further investigation, including clinical cases treated with combination therapy.

Because lower serum sodium levels have been associated with increased mortality and worse outcomes in patients with mRCC treated with TKIs^18,19,26,34,35^ and in light of our findings, sodium level could serve as further indicator for a better risk stratification and therapy selection, especially in the context of combination therapies. Furthermore, considering the previously reported negative role of lower sodium levels in the preoperative setting,^19,36^ our findings may contribute to improved management of patients with localized RCC eligible for adjuvant ICI treatment.

### Limitations

This study has several limitations, primarily because of its retrospective nature and the use of second-line therapy that is no longer considered the standard of care, except for selected cases (eg, patients with favorable IMDC scores). Second, patients’ comorbidities and their specific concomitant medications, especially antihypertensive drugs, were not assessed. Moreover, several factors, such as the conditions at the time of sampling or having undergone nephrectomy, could have influenced the sodium levels in these patients. In addition, although only 26 patients in our analysis had pre-ICI sodium levels below the lower limit of the laboratory range (≤135 mEq/L), they may have had a greater impact on the poor outcomes. For these patients, it might be worth considering interventions to increase sodium levels to normal values, although no data are available in the literature regarding this approach. Despite these limitations, the study’s strengths include multicenter involvement, a large number of patients included, and the evaluation of natremia at baseline and after initiating treatment.

## Conclusions

In conclusion, our study revealed that in patients with mRCC receiving nivolumab as a second-line or subsequent line treatment, a pre-ICI serum sodium level greater than or equal to 140 mEq/L was associated with longer OS compared with sodium levels less than 140 mEq/L. Patients with sodium levels greater than or equal to 140 mEq/L after initiating treatment had better PFS, OS, and DCR compared with those with levels greater than or equal to 140 mEq/L. In addition, patients with at least a sodium value greater than or equal to 140 mEq/L at pre-ICI and post-ICI had higher OS and PFS and longer DCR compared with patients with sodium levels greater than or equal to 140 mEq/L for both time points. To the best of our knowledge, higher sodium levels may serve as an important factor associated with better survival outcomes in patients with RCC receiving immunotherapy, suggesting its potential use as an additional variable to consider in patients’ risk scores. Further and prospective studies are needed to confirm our findings, especially in patients who currently can receive combinations based on immunotherapy.

## Notes

Supplement 1. **eTable 1.** Patients’ Characteristics According to Pre-ICI Sodium Value **eTable 2.** Patients’ Characteristics According to Post-ICI Sodium Value **eTable 3.** Patients’ Characteristics According to Pre- and Post-ICI Sodium Value **eTable 4.** Best Response, PFS and OS According to Serum Sodium Values

Supplement 2.
Data Sharing Statement
