# Supplementary material for: Sodium Levels and Outcomes in Patients With Metastatic Renal Cell Carcinoma Receiving Nivolumab
Source: JAMA Netw Open. 2023 Nov 27;6(11):e2345185. doi: 10.1001/jamanetworkopen.2023.45185 (PMC10682835; doi:10.1001/jamanetworkopen.2023.45185)
Supplement: Supplement 1. — eTable 1. Patients’ Characteristics According to Pre-ICI Sodium Value eTable 2. Patients’ Characteristics According to Post-ICI Sodium Value eTable 3. Patients’ Characteristics According to Pre- and Post-ICI Sodium Value eTable 4. Best Response, PFS and OS According to Serum Sodium Values [file jamanetwopen-e2345185-s001.pdf]

Supplemental Online Content

Catalano M, Rebuzzi SE, Maruzzo M, et al. Sodium levels and outcomes in patients with metastatic renal cell carcinoma receiving nivolumab. *JAMA Netw Open*. 2023;6(11):e2345185. doi:10.1001/jamanetworkopen.2023.45185

eTable 1. Patients' Characteristics According to Pre-ICI Sodium Value

eTable 2. Patients' Characteristics According to Post-ICI Sodium Value

eTable 3. Patients' Characteristics According to Pre- and Post-ICI Sodium Value

eTable 4. Best Response, PFS and OS According to Serum Sodium Values

This supplemental material has been provided by the authors to give readers additional information about their work.

© 2023 Catalano M et al. *JAMA Network Open.*

eTable 1. Patients' Characteristics According to Pre-ICI Sodium Value

	**≥140 mEq/L (n=194)**	**<140 mEq/L (n=161)**	***p***
**Age**			
Median (range)	75 (45-84)	75 (44-83)	0.90
**Gender,** n (%)			
Male	138 (71.1)	120 (74.5)	0.51
**Histology,** n (%)			
Clear-cell RCC	166 (86.0)	140 (87.5)	0.70
**Previous nephrectomy,** n (%)			
Yes	170 (87.6)	143 (88.8)	0.72
**Karnofsky-PS,** n (%)			
≥80%	174 (89.7)	184 (83.2)	0.12
**IMDC score,** n (%)			
Intermediate-poor	150 (77.3)	129 (80.1)	0.60
**Sites of metastases,** n (%)			
Lymph-nodal	108 (55.7)	94 (58.4)	0.60
Visceral	175 (90.2)	151 (93.8)	0.21
Bone	69 (35.6)	51 (31.7)	0.50
**First-line therapy,** n (%)			
Sunitinib	121 (64.0)	99 (45)	0.82
Pazopanib	68 (53.5)	59 (46.5)	
**Nivolumab line,** n (%)			
Second line	135 (55.1)	110 (44.9)	0.80
⩾Third line	59 (53.6)	51 (41.6)	

RCC: renal cell carcinoma; IMDC: international metastatic renal cell carcinoma database consortium; ICI: immune checkpoint inhibitor; PS: performance status.

© 2023 Catalano M et al. *JAMA Network Open.*

eTable 2. Patients' Characteristics According to Post-ICI Sodium Value

	**≥140 mEq/L (n=172)**	**<140 mEq/L (n=183)**	***p***
**Age**			
Median (range)	75 (44-81)	75 (45-84)	0.80
**Gender,** n (%)			
Male	121 (70.3)	137 (74.9)	0.31
**Histology,** n (%)			
Clear-cell RCC	150 (87.7)	156 (85.7)	0.62
**Previous nephrectomy,** n (%)			
Yes	151 (87.8)	162 (88.5)	0.90
**Karnofsky-PS,** n (%)			
≥80%	155 (90.1)	153 (83.6)	0.11
**IMDC score,** n (%)			
Intermediate-poor	134 (77.9)	145 (79.2)	0.80
**Sites of metastses,** n (%)			
Lymph-nodal	91 (52.9)	111 (60.7)	0.22
Visceral	156 (90.7)	170 (92.9)	0.60
Bone	56 (32.6)	64 (35.0)	0.61
**First-line therapy,** n (%)			
Sunitinib	104 (62.6)	116 (64.1)	0.80
Pazopanib	62 (37.3)	65 (35.9)	
Other			
**Nivolumab line,** n (%)			
Second line	121 (70.3)	124 (67.8)	0.80
⩾Third line	51 (29.7)	59 (32.2)	

RCC: renal cell carcinoma; IMDC: international metastatic renal cell carcinoma database consortium; ICI: immune checkpoint inhibitor; PS: performance status.

© 2023 Catalano M et al. *JAMA Network Open.*

eTable 3. Patients' Characteristics According to Pre- and Post-ICI Sodium Value

	≥140 mEq/L (n=122)	<140 mEq/L (n=233)	*p*
**Age**			
Median (range)	75 (54-81)	75 (44-84)	0.81
**Gender,** n (%)			
Male	86 (70.5)	172 (73.8)	0.50
**Histology,** n (%)			
Clear-cell RCC	104 (85.9)	202 (87.1)	0.70
**Previous nephrectomy** n (%)			
Yes	106 (86.9)	207 (88.8)	0.62
**Karnofsky-PS,** n (%)			
≥80%	113 (92.6)	195 (83.7)	0.12
**IMDC score,** n (%)			
Intermediate-poor	92 (75.4)	187 (80.3)	0.30
**Sites of metastases,** n (%)			
Lymph-nodal	66 (54.1)	136 (58.4)	0.50
Visceral	109 (89.3)	217 (93.1)	0.61
Bone	41 (33.6)	64 (35.0)	0.60
**First-line therapy,** n (%)			
Sunitinib	74 (62.7)	146 (63.8)	0.91
Pazopanib	44 (37.3)	83 (36.2)	
**Nivolumab line,** n (%)			
Second line	84 (68.8)	161 (69.1)	0.92
⩾Third line	38 (31.2)	72 (30.9)	

RCC: renal cell carcinoma; IMDC: international metastatic renal cell carcinoma database consortium; TKI: tyrosine kinase inhibitor.

© 2023 Catalano M et al. *JAMA Network Open.*

eTable 4. Best Response, PFS and OS According to Serum Sodium Values

	**ORR** n, (%)	**DCR** n, (%)	**Median PFS** months, (95% CI)	**Median OS** months, (95% CI)
**All patients (n=355)**	114 (32.1)	213 (60)	8.3 (6.4-10.2)	25.1 (20.2-29.9)
**Pre-ICI Na**				
≥140 mEq/L (n=194)	58 (29.9)	124 (63.9)	9.3 (6.5-11.5)	29.2 (21.8-35.9)
<140 mEq/L (n=161)	56 (34.8)	89 (55.3)	7.4 (4.6-10.1)	20 (14.1-26.8)
	P=.20	*P*=.10	*P*=.90	*P*=.03
**Post-ICI Na**				
≥140 mEq/L (n=172)	60 (34.9)	120 (69.8)	11.1 (8.5-15)	32.9 (25.1-42.6)
<140 mEq/L (n=183)	54 (29.5)	93 (50.8)	5.1 (4.1-7.5)	17.1 (12.6-24.5)
	P=.20	*P*=.006	*P*=.01	*P*=.006
**Pre-and post-ICI Na**				
≥140 mEq/L (n=122)	40 (32.8)	87 (71.3)	11.5 (8.8-16.4)	37.6 (29-49.9)
<140 mEq/L (n=233)	74 (31.8)	126 (54.1)	5.8 (4.4-8.3)	19.4 (14.1-24.5)
	P=.50	*P*=.004	*P*=.008	*P*=.01

ORR: objective response rate; DCR: disease control rate; PFS: progression free survival; OS: overall survival; Na: serum sodium; CI: confidence interval; ICI: immune checkpoint inhibitor.

© 2023 Catalano M et al. *JAMA Network Open.*
