# Supplementary material for: Sodium Levels and Outcomes in Patients With Metastatic Renal Cell Carcinoma Receiving Nivolumab
Source: JAMA Netw Open. 2023 Nov 27;6(11):e2345185. doi: 10.1001/jamanetworkopen.2023.45185 (PMC10682835; doi:10.1001/jamanetworkopen.2023.45185)
Supplement: Supplement 2. — Data Sharing Statement [file jamanetwopen-e2345185-s002.pdf]

Data Sharing Statement

Catalano. Sodium Levels and Outcomes in Patients With Metastatic Renal Cell Carcinoma Receiving Nivolumab. *JAMA Netw Open*. Published November 27, 2023.
doi:10.1001/jamanetworkopen.2023.45185

Data
Data available: No

Additional Information
Explanation for why data not available: The data will be provide on required
